# Targeted Degradation of eEF2K by a Structure‐Guided PROTAC Strategy for the Treatment of Triple‐Negative Breast Cancer

**DOI:** 10.1002/advs.202520863

**Published:** 2026-01-27

**Authors:** Shijun Cao, Changxin Zhong, Shilong Jiang, Yungui Li, Yang Xi, Mingxuan Xiao, Ting Jiang, Xiaoya Wan, Zonglin Chen, Xiaohui Yu, Yan Cheng

**Affiliations:** ^1^ Department of Pathology The Affiliated Cancer Hospital of Xiangya School of Medicine Central South University /Hunan Cancer Hospital Changsha Hunan China; ^2^ Department of Pharmacy The Second Xiangya Hospital Central South University Changsha Hunan China; ^3^ Hunan Provincial Engineering Research Centre of Translational Medicine and Innovative Drug Changsha Hunan China; ^4^ Department of Pharmacy Xiangya Hospital Central South University Changsha Hunan China; ^5^ The Hunan Institute of Pharmacy Practice and Clinical Research Xiangya Hospital Central South University Changsha Hunan China; ^6^ Department of General Surgery The Second Xiangya Hospital Central South University Changsha Hunan China; ^7^ NHC Key Laboratory of Cancer Proteomics & State Local Joint Engineering Laboratory for Anticancer Drugs Xiangya Hospital Central South University Changsha Hunan China; ^8^ Clinical Research Center for Breast Disease in Hunan Province Changsha Hunan China; ^9^ FuRong Laboratory Changsha Hunan China

**Keywords:** A6, A6@ZIF‐8, eEF2K, PROTAC, Triple‐negative breast cancer

## Abstract

Proteolysis‐targeting chimera (PROTAC) have emerged as a promising class of anticancer agents. Eukaryotic elongation factor 2 kinase (eEF2K), a stress‐responsive regulator of translational elongation, has emerged as a pivotal therapeutic target in triple‐negative breast cancer (TNBC) due to its critical role in sustaining cancer cell survival under nutrient stress. Building on our previous work identifying eEF2K as an oncogenic kinase, this study developed an eEF2K‐targeting PROTAC that exhibited potent antitumor activity against TNBC. Through a structure‐guided design, we identified a key allosteric pocket of eEF2K and linked its inhibitor 2S to a CRBN ligand to generate A6, a PROTAC that promotes eEF2K degradation via a conformationally optimized interface, achieving >90% target depletion while preserving the total levels of its substrate eEF2. A6 exhibited anti‐proliferative effects across TNBC cell lines by degrading eEF2K. In Vivo and in TNBC organoid models, A6 treatment significantly suppressed tumor growth, with favorable tolerability. To further enhance tumor‐specific delivery, we engineered A6@ZIF‐8, a pH‐sensitive nanocarrier that promotes drug accumulation at tumor sites compared to free A6, leading to improved therapeutic outcomes. Collectively, our data indicate that targeted degradation of eEF2K via PROTAC technology constitutes a novel and therapeutically relevant intervention strategy for TNBC.

## Graphical abstract

This study developed an eEF2K‐targeting PROTAC, A6, that efficiently degrades eEF2K in TNBC cells, inhibiting tumor growth in vitro and in vivo. To enhance tumor‐specific delivery, we engineered A6@ZIF‐8, a pH‐sensitive nanocarrier, which improved drug accumulation at tumor sites, offering a promising therapeutic strategy for TNBC through targeted degradation of eEF2K.

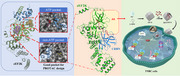

## 1 Introduction

Triple‐negative breast cancer (TNBC), an aggressive subtype defined by the absence of estrogen receptor (ER), progesterone receptor (PR), and human epidermal growth factor receptor 2 (HER2) expression [1, 2], presents significant clinical challenges. The lack of these targetable receptors renders conventional endocrine therapies and HER2‐targeted agents ineffective, directly contributing to its poor prognosis and limited therapeutic options [3]. These unmet needs underscore the urgency of identifying TNBC‐specific molecular vulnerabilities and developing mechanistically targeted therapies [4].

Eukaryotic elongation factor 2 kinase (eEF2K), a noncanonical member of the α‐kinase family, critically regulates translation by phosphorylating its substrate, eEF2 [5]. Emerging studies highlight the pathological overexpression of eEF2K across various malignancies, which strongly correlates with poor clinical outcomes [6]. This kinase has been reported to promote tumor progression by enhancing cancer cell survival, proliferation, metastasis, and therapy resistance [7, 8, 9, 10, 11]. Recent evidence further indicates that eEF2K inhibition activates the cGAS‐STING pathway, thereby enhancing natural killer (NK) cell infiltration and cytotoxic activity in hepatocellular carcinoma (HCC) models [12]. In TNBC specifically, eEF2K upregulation directly contributes to aggressive oncogenic phenotypes, including hyperproliferation, enhanced migration, and increased invasiveness. Consequently, targeting eEF2K represents a promising therapeutic strategy for TNBC [13, 14].

Our previous research has established eEF2K as a key regulator of autophagy flux [15, 16, 17], aerobic glycolysis [18], multidrug resistance [19, 20], and the exclusion of immunosuppressive T cells from tumor microenvironments [21]. Using artificial intelligence, we identified mitoxantrone as a potential eEF2K inhibitor that enhances the antitumor efficacy of mTOR inhibitors [19]. Moreover, we developed molecular glues targeting eEF2K, which exhibit significant antitumor activity in triple‐negative breast cancer (TNBC) [20, 22, 23]. These findings reveal novel therapeutic vulnerabilities linked to eEF2K's diverse functions, paving the way for precision targeting strategies to overcome current challenges in cancer therapy. Although recent drug discovery efforts have produced several small molecules targeting eEF2K, existing inhibitors are hampered by either limited efficacy (e.g., A‐484954) or poor selectivity (e.g., NH125) [6, 24, 25]. This underscores the urgent need for the developing novel therapeutic approaches targeting eEF2K for cancer treatment.

In recent years, targeted protein degradation (TPD) has revolutionized drug discovery by shifting the focus from functional inhibition to the proteasome‐mediated elimination of disease‐causing proteins, particularly addressing the “undruggable” proteome by hijacking the ubiquitin‐proteasome system [26, 27]. Small molecule PROTAC degraders offer distinct advantages over traditional kinase inhibitors, and have garnered significant attention for their potential in cancer therapy [28]. Unlike conventional inhibitors, PROTACs do not require selective binding ligands and operate substoichiometrically, enabling sustained pharmacological effects at lower doses [29, 30]. Moreover, PROTAC‐mediated kinase degradation is a catalytic, event‐driven process, reducing the likelihood of resistance mutations compared to occupancy‐driven inhibition [31]. Therefore, developing eEF2K‐targeting PROTACs holds significant promise for cancer therapy, offering a strategy to achieve selective eEF2K degradation and enhance antitumor efficacy. However, despite these compelling theoretical advantages, the development of eEF2K‐targeted PROTACs remains nascent. To date, only one study has explored CRBN‐recruiting PROTACs based on the scaffold of A‐484954, achieving suboptimal degradation efficiency (56.7% maximum) and limited antitumor efficacy [32].

In this study, we developed A6, a small‐molecule PROTAC degrader targeting eEF2K, and demonstrated its significant anti‐TNBC activity *in vitro, in vivo*, and in patient‐derived TNBC organoids. To enhance PROTAC delivery, we engineered an actively targeted nanomedicine system using metal‐organic frameworks (MOFs), fabricating A6@ZIF‐8 for efficient in vivo delivery. Our findings suggest that PROTAC‐mediated eEF2K degradation represents a novel therapeutic avenue for TNBC, with significant therapeutic implications.

## 2 Methods and Materials

### 2.1 Ethics Approval

The study protocol involving human participants was reviewed and approved by the Medical Ethics Review Committee of the Second Xiangya Hospital, Central South University (No. LYEC2025‐K0011). All human tissue samples were collected from the Second Xiangya Hospital, Central South University (Changsha, China) with written informed consent provided by each participant prior to sample collection.

All animal experiments were approved by the Institutional Animal Care and Use Committee (IACUC) of Central South University (No.CSU‐2022‐0013) and were conducted incompliance with institutional guidelines.

### 2.2 Cell Culture

BT549 and HCC1806 cells were purchased from the Cell Bank of the Chinese Academy of Sciences (Shanghai, China) in 2022, while MDA‐MB‐231 cells were purchased from the Cell Bank of the Chinese Academy of Sciences (Beijing, China) in 2020. All cell lines were authenticated by short tandem repeat (STR) profiling and routinely tested negative for mycoplasma contamination using a commercial detection kit. BT549 and HCC1806 cells were maintained in RPMI‐1640 medium supplemented with 10% fetal bovine serum, while MDA‐MB‐231 cells were cultured in DMEM with 10% FBS and 1% Penicillin‐Streptomycin Solution under standard conditions (37°C, 5% CO_2_).

### 2.3 Reagent and Antibodies

Fetal Bovine Serum (Cat# FND500) was sourced from ExCell Bio (Shanghai, China), and SDS‐PAGE Loading Buffer (Cat# WB2001) from New Cell & Molecular Biotech (Suzhou, China). MLN4924 (Cat# T6332) was purchased from Targetmol (USA), while MG132 (Cat# S2619) were obtained from Selleck (www.selleckchem.com, Houston, TX, USA). The anti‐eEF2K antibody (Cat# ab85721) was sourced from Abcam (Cambridge, UK). Antibodies against GAPDH (Cat# GB11002) and Ki‐67 (Cat# GB111499) were purchased from Servicebio (Wuhan, China). Antibodies targeting Bcl‐2 (Cat# 15071) were obtained from Cell Signaling Technology (Danvers, MA, USA). Antibodies against N‐Cadherin (Cat# ET1607‐37), Vimentin (Cat# M1412‐1), and E‐Cadherin (Cat# ET1607‐75) were from HUABIO (Hangzhou, China). Anti‐Bax (Cat# 50599‐2‐Ig) antibodies were purchased from Proteintech (Chicago, IL, USA).

### 2.4 Cell Viability Assay

Cell viability was assessed using the CCK‐8 reagent (Bimake, TX, USA). Briefly, cells were plated in a 96‐well plate and treated with varying concentrations of the drug. Following treatment, 10 µL of CCK‐8 reagent was added to each well, and the cells were incubated for 1–2 h. Absorbance was measured at a wavelength of 450 nm.

### 2.5 Colony‐Formation Assay

MDA‐MB‐231, HCC1806, or BT549 cells were seeded into six‐well plates and treated with different concentrations of the drug for two days, with media replaced every 2–3 days. Afterward, cells were stained with 0.5% crystal violet (Beyotime Biotechnology, Shanghai, China) and washed with PBS, and the plates were photographed.

### 2.6 Wound‐Healing Assay

Cells were seeded in a six‐well plate and a wound was created by scratching the monolayer with a pipette tip when the cells reached 90%‐100% confluence. Cells were then exposed to the drug for 48 h. Cell migration was monitored using microscopy and images were captured at 0 and 48 h.

### 2.7 Flow‐Cytometric Analysis of Apoptosis

For apoptosis analysis, cells were washed twice with cold PBS, resuspended in 5 µL Annexin V‐FITC and 10 µL propidium iodide (PI) staining buffer (BD), and incubated in the dark for at least 15 min. The stained cells were analyzed by FACS.

### 2.8 Plasmids Transfection

For transfection, Flag‐eEF2K, Myc‐Ub, and sheEF2K were incubated with Lipofectamine 8000 (Beyotime Biotechnology) in a serum‐free DMEM medium, then added to the cells. Protein expression was assessed 48 h post‐transfection.

### 2.9 Mouse Xenograft Model

All animal experiments were approved by the Institutional Animal Care and Use Committee (IACUC) of Central South University, in line with the welfare and ethical principles of laboratory animals. MDA‐MB‐231 cells (1 × 10^6^/mouse) were suspended in 100 µL DMEM medium and injected subcutaneously into the right flank of 4‐week‐old female nude mice. Each group consisted of 6 mice. Tumor‐bearing mice were randomized into treatment groups when tumors reached 80–90 mm^3^ (Volume = length × width^2^ × π/6). Treatment lasted for 14 days, with DMSO, 2S, A6, or A6‐Me injected intraperitoneally every other day. The groups were treated as follows: (1) vehicle (DMSO, i.p.), (2) 2S (20 mg/kg, i.p.), (3) A6 (10 mg/kg, i.p.), (4) A6 (20 mg/kg, i.p.), (5) A6‐Me (20 mg/kg i.p.). For A6@ZIF‐8‐treated experiments, mice were randomized into three groups and treated with: (1) vehicle (saline, i.p.), (2) A6 (2.5 mg/kg, i.p.), (3) A6@ZIF‐8 (2.5 mg/kg, i.p.).

### 2.10 Immunohistochemistry (IHC)

Formalin‐fixed, paraffin‐embedded tumor tissues were sectioned at 4–5 µm thickness. Sections were deparaffinized, rehydrated through a graded ethanol series, and subjected to antigen retrieval according to standard protocols. Endogenous peroxidase activity was blocked with 3% hydrogen peroxide, followed by incubation with blocking solution to reduce non‐specific binding. The sections were then incubated overnight at 4°C with primary antibodies against Ki‐67 and eEF2K, following the manufacturer's instructions. After washing, sections were incubated with appropriate secondary antibodies and visualized using a DAB chromogen kit. Finally, sections were counterstained with hematoxylin, dehydrated, and mounted for microscopic evaluation.

### 2.11 Blood Samples and Analyses for In Vivo Toxicity

Serum biochemical parameters for kidney and liver were measured for each animal by the Department of Laboratory Medicine, The Second Xiangya Hospital, Central South University. These parameters included blood urea nitrogen (UREA), aspartate aminotransferase (AST), alanine aminotransferase (ALT), creatinine (CREA), lactic dehydrogenase (LDH), and AST / ALT.

### 2.12 Western Blotting Analysis

After the indicated treatments, cells were lysed with RIPA buffer (Beyotime Biotechnology, Shanghai, China) containing a protease inhibitor cocktail and phosphatase inhibitors. The cell lysate was then centrifuged at 12 000 rpm at 4°C for 15 min. Protein concentration in the supernatant was quantified using the Bradford assay (Beyotime Biotechnology, Shanghai, China). Subsequently, 20 µg of protein was loaded onto an SDS‐PAGE gel and transferred to a 0.2 µm PVDF membrane. The membranes were blocked with 5% skim milk for 1 h at room temperature, incubated with primary antibody overnight at 4°C, followed by secondary antibody incubation for 1 h at room temperature. Protein signals were detected using an enhanced chemiluminescence kit.

### 2.13 Cellular Thermal Shift Assay (CETSA)

Breast cancer cells were treated with or without A6 overnight. After treatment, cells were collected, and equal volumes of cell suspensions were heated at varying temperatures for 3 min, followed by cooling to room temperature for an additional 3 min. Cells were then lysed by three freeze‐thaw cycles in liquid nitrogen. Lysates were centrifuged at 15 000 g for 15 min at 4°C, and the supernatant was used for SDS‐PAGE gel and western blotting analysis.

### 2.14 Molecular Docking and Molecular Dynamics Simulation

Docking simulations between 2S and eEF2K (PDB ID: 8gm4) were performed using Schrödinger software (2021‐4, LLC, New York, NY, USA). Additionally, the software was employed to simulate the binding interactions between A6, eEF2K, and CRBN (PDB ID: 4v2z) in ternary complexes, along with molecular dynamics (MD) studies.

### 2.15 NanoBRET Assay

Transient transfection of the dual NanoLuc and Halo‐tagged CRBN Tandem plasmid (Promega) was performed as previously described. Briefly, 9.0 µg of the DDB1 Expression Vector and 1.0 µg of NanoLuc‐CRBN plasmid were mixed in 1 mL of Opti‐MEM, followed by the addition of FuGENE HD (Promega, N2910, USA) at a 30:1 ratio. This mixture was then added to each well of a 6‐well plate seeded with HEK293 cells at 75% confluence. The following morning, the medium was replaced with phenol red‐free Opti‐MEM (Gibco) containing or lacking 0.1 mM HaloTag NanoBRET ligand (Promega), while shaking at 900 RPM for 15 s. Subsequently, A6 or A6@ZIF‐8 was added while shaking at 900 RPM for 15 s. After incubating for 3 min in the presence of complete Substrate Plus Inhibitor Solution (Promega), the plate was placed in darkness for 3 h. Multimodal enzyme labelers were used to detect signals at 610 and 450 nm.

### 2.16 Cell Intake Assay

MDA‐MB‐231 cells were seeded in a 6‐well plate at a density of 10^7^ cells/mL and treated with A6 or A6@ZIF‐8 at the same concentrations (1 and 2 µM). After a 12 h incubation at 37°C to reach a steady state, the medium was discarded, and the cells were washed twice with PBS. The cells were then collected in EP tubes, sonicated for 2 min at 20 W with a cell sonicator, and the supernatants were collected. Intracellular accumulation of A6 was measured by HPLC.

### 2.17 Preparation of A6@ZIF‐8 NPs

To prepare A6@ZIF‐8 nanoparticles (NPs), 10 µL of 10 mM A6 in DMSO was rapidly added to 1 mL of 60 mM 2‐methylimidazole (2‐MIM) solution under vigorous stirring, which was continued at room temperature for 5 min. Then, 1 mL of 10 mM zinc nitrate hexahydrate solution was added dropwise, and stirring was maintained at room temperature. After the reaction, the mixture was centrifuged at 16 000 rpm, the supernatant was discarded, and the precipitate was redispersed in deionized water to obtain A6@ZIF‐8 NPs.

### 2.18 Characterization of A6@ZIF‐8 NPs

The particle size and zeta potential of A6@ZIF‐8 NPs were measured using a Malvern Zetasizer Nano series (Nano ZS, Malvern Instruments) via dynamic light scattering (DLS). The morphology of the NPs was observed using transmission electron microscopy and energy‐dispersive spectroscopy (TEM‐EDS, Titan G2 60–300, FEI).

### 2.19 Drug Loading Efficiency

After nanoparticle formation, A6@ZIF‐8 nanoparticles were collected by centrifugation (16 000 rpm, 15 min) and washed 3 times to remove free A6. The nanoparticles were freeze‐dried to obtain the dry mass. A known amount of dried A6@ZIF‐8 was then completely digested in acid to dissociate the ZIF‐8 framework and release encapsulated A6. The resulting solution was filtered (0.22 µm) and analyzed by HPLC. The amount of A6 was calculated using the following formula:

$$Drug\;loading\;efficiency=\frac{W_{a6-loaded}}{W_{A6@\mathrm{ZIF}-8\;NPs}}\times 100\%$$

### 2.20 Encapsulation Efficiency

A6 was loaded into ZIF‐8 NPs by mixing an aqueous solution of A6 with 2‐MIM and adding it dropwise to an aqueous solution of zinc nitrate hexahydrate. The amount of A6 loaded in the A6@ZIF‐8 NPs was quantified by HPLC, where the amount of A6 remaining in the supernatant was subtracted from the total mass of A6. The encapsulation efficiency was calculated using the following formula:

$$Encapsulation\;efficiency=\frac{a6_{Added}-a6_{Supernatant}}{a6_{Added}}\times 100\%$$

### 2.21 Cellular Uptake Assay of A6@ZIF‐8 NPs

MDA‐MB‐231 cells were seeded in a 35 mm glass‐bottom Petri dish. After incubation with A6@ZIF‐8/6‐FAM for a specified time, the cells were washed twice with PBS. Phalloidin (0.1 µM) was then added to mark the cytoskeleton and incubated for 30 min, while nuclei were stained with DAPI (1 µg/mL) for 8 min. The cells were imaged using a fluorescence microscope (OLYMPUS IX73, Japan).

### 2.22 In Vivo/Vitro Fluorescence Imaging

Animal protocols were in compliance with the institutional guidelines of the Animal Care and Use Committee of Central South University. MDA‐MB‐231 cells, suspended in 100 µL PBS, were subcutaneously injected into the axilla of each female BALB/c nude mouse. When the tumors reached approximately 100 mm^3^, mice were treated with different formulations. For biodistribution studies, mice were intravenously injected with either free A6 or A6@ZIF‐8 NPs and imaged at designated time points post‐injection using a PerkinElmer in vivo optical imaging system (IVIS Lumina). Mice were euthanized 24 h post‐injection, and major organs (heart, liver, spleen, lung, and kidney) along with tumors were collected for ex vivo imaging. Images were analyzed using Living Imaging software.

### 2.23 Hemolysis Assay of A6@ZIF‐8 NPs

Hemolysis assays were performed to assess in vitro biocompatibility. Fresh mouse blood (5 mL) was collected and stabilized with 0.2 mL heparin. The blood sample was mixed with 5 mL of 0.9% saline and centrifuged at 5000 rpm for 5 min. The supernatant was discarded, and the red blood cells (RBCs) were washed five times and resuspended in saline to a 2% concentration. The RBC suspension (0.5 mL) was then mixed with (a) 0.5 mL saline (negative control), (b) 0.5 mL ultrapure water (positive control), and (c) 0.5 mL A6@ZIF‐8 NPs suspension at concentrations ranging from 50 to 1000 µg/mL. The samples were incubated at 37°C for 30 min, followed by centrifugation at 3000 rpm for 10 min. The absorbance of the supernatant at 540 nm was measured using a microplate reader (Synergy 4, BioTek). The hemolysis rate was calculated as follows:

$$Hemolysis\;rate=\frac{A_{Sample}-A_{Negative}}{A_{Positive}-A_{Negative}}\times 100\%$$

### 2.24 Drug Release of A6@ZIF‐8 NPs

To mimic the microenvironments of normal and tumor tissues, PBS at pH 7.4, 6.5, and 5.0 was employed. The tumor microenvironment (TME)‐responsive drug release behavior of A6@ZIF‐8 NPs was assessed by placing 5 mL of the A6@ZIF‐8 NPs aqueous solution into a dialysis bag (molecular weight cut‐off: 3 kDa) and immersing it in the respective media, with incubation in a shaking incubator at 37°C. At designated time intervals, 1 mL of the dialysis fluid was withdrawn, and a fresh medium was added to replace the removed volume. The free A6 released from A6@ZIF‐8 was quantified using a CAPCELL PAK C18 column (250 mm × 4.6 mm, 5.0 µm).

### 2.25 Patient‐Derived Organoid

TNBC tissue‐derived organoids were established as described previously [33, 34, 35]. Briefly, tumor specimens were digested with collagenase (Sigma) to generate single‐cell suspensions, which were resuspended in culture medium and embedded in 50% chilled Matrigel (Corning) to enable three‐dimensional growth. After 5 days, organoids were dissociated into single cells and seeded into 96‐well plates. The cultures were then treated with A6 or A6@ZIF‐8, and fluorescence microscopic imaging was performed to assess tumor cell proliferation.

### 2.26 Stability Assay of A6@ZIF‐8 NPs

The synthesized NPs were dispersed separately in deionized water, physiological saline, FBS, and complete DMEM culture medium, and incubated at room temperature. Particle size and colloidal stability of the nanoformulation were assessed by collecting samples at 0, 0.25, 0.5, 1, 2, 3, and 4 days.

### 2.27 Statistical Analysis

Data analysis for all experiments was performed using GraphPad Prism (version 8.0.1). Data are presented as the mean ± standard deviation (SD) from three or more independent experiments. Two group comparisons were analyzed using the *t*‐test, and for multiple group comparisons, ANOVA was used to calculate *p*‐values. Statistical significance was determined for *p* < 0.05 (*, *p* < 0.05; **, *p* < 0.01; ***, *p* < 0.001).

## 3 Results

### 3.1 Rational Design of Cereblon‐Recruiting eEF2K Degraders

**FIGURE 1:**
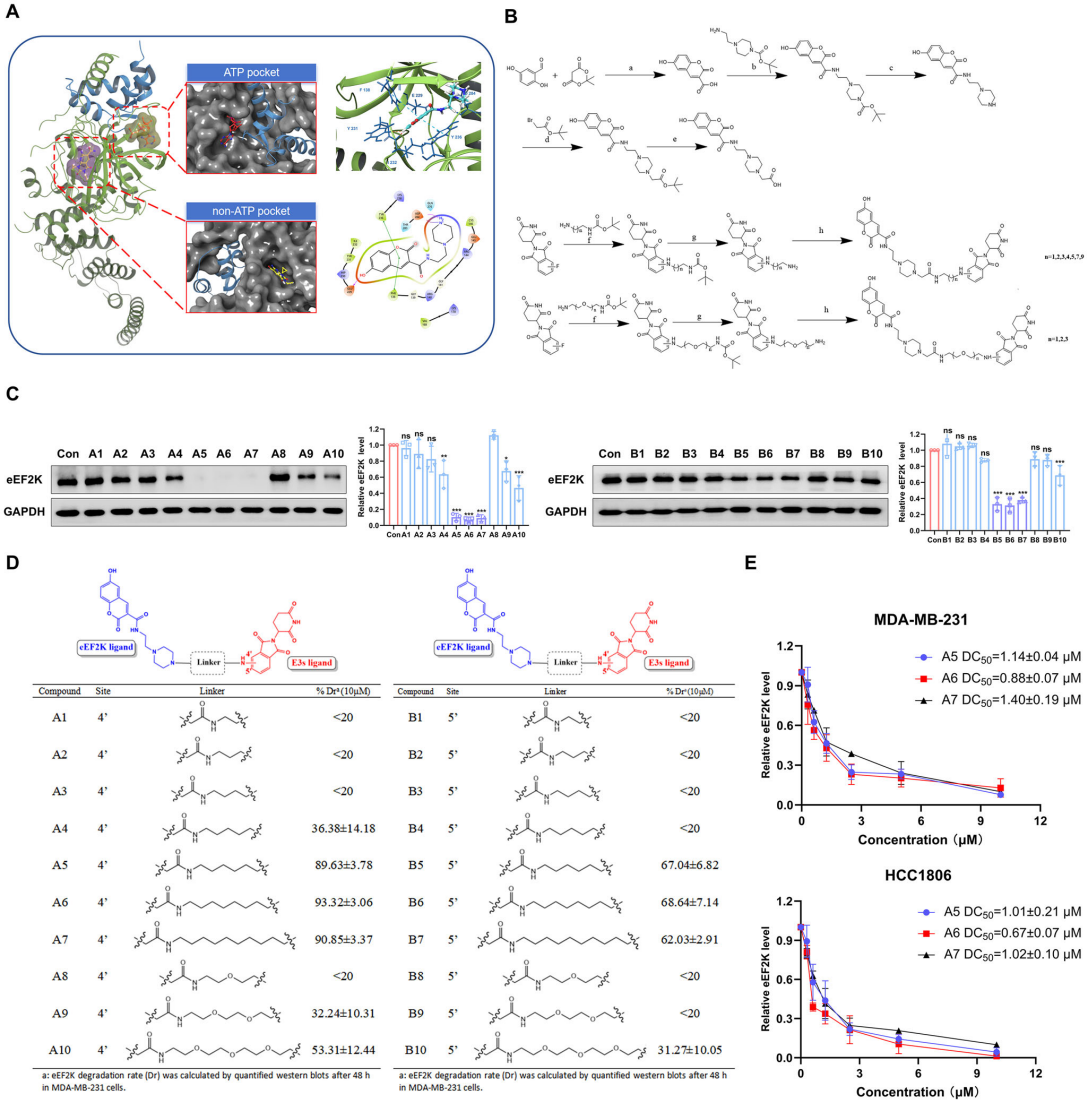
Rational design of cereblon‐recruiting eEF2K degraders. (A) The complex formed between eEF2K (green ribbon) and calmodulin (blue ribbon) (PDB code: 8gue), showing the binding modes of ADP (red atom‐colored stick) in the ATP Pocket and A484954 (yellow atom‐colored stick) in the non‐ATP Pocket. (B) Synthesis of eEF2K degraders. Reagents and conditions: a) 75°C, water, 4 h; b) HOBT, EDCI, TEA, DMF (dry), 6 h; c) CF_3_COOH, DCM, 1 h; d) HOBT, EDCI, TEA, DMF (dry), 6 h; e) CF_3_COOH, DCM, 1 h; f) DIPEA, DMF (dry), overnight; g) CF_3_COOH, DCM, 1 h; h) HOBT, EDCI, TEA, DMF (dry), 6 h. (C) eEF2K degradation in MDA‐MB‐231 cells treated with PROTACs (10 µM, 48 h). Data are presented as mean ± SD. *p < 0.05, **p < 0.01, ***p < 0.001, ns, *p* > 0.05. (D) Degradation efficiency quantification from panel C. Statistical analysis performed with triplicate experiments. (E) Antiproliferative effects of A5‐A7 in breast cancer cells (72 h treatment). DC_50_ values were derived from CCK‐8 assays. Error bars represent SD (n = 3).

Structural analysis of eEF2K (PDB ID: 8GM4) identified two ligand‐binding sites: a canonical ATP‐binding pocket and an allosteric pocket critical for substrate recognition. To avoid off‐target effects associated with ATP‐competitive inhibition, we prioritized the allosteric site for PROTAC development. Molecular docking simulations (Glide XP) using the known allosteric binder 2S revealed optimal binding geometry (ΔG = ‐46.943 kcal/mol), stabilized by π‐π stacking with F138 and hydrogen bonding to E229 (Figure 1A). This validated 2S as the ideal warhead for PROTAC conjugation. Given the established utility of cereblon (CRBN) in PROTAC design and thalidomide's ability to recruit CRBN via its IMiD pharmacophore [36, 37], we designed and synthesized a library of chimeric degraders by conjugating the eEF2K‐targeting warhead 2S to thalidomide derivatives via polyethylene glycol (PEG) linkers (Figure 1B). Screening for eEF2K degradation activity identified compounds A5‐A7 and B5‐B7 as the most effective degraders, with their degradation rates (Dr) quantitatively assessed against control treatments (Figure 1C,D). Furthermore, A6 exhibited superior antiproliferative activity (IC_50_ = 13.98 µM in MDA‐MB‐231; 8.70 µM in HCC1806)compared to other derivatives (Table S1). We also investigated the inhibitory effects of A6 on different subtypes of breast cancer cells, and demonstrated that A6 effectively suppresses the proliferation of various breast cancer subtypes, such as ER (+) breast cancer cell lines (MCF‐7 and T47D) and HER2 (+) breast cancer cell lines (SK‐BR‐3), while exerting only minimal cytotoxicity toward normal breast epithelial cells (MCF‐10A) (Table S2). Consistent with its potency, A6 achieved the lowest DC_50_ (half‐maximal degradation concentration) in cellular degradation assays (Figure 1E). Based on its dual efficacy in degradation and growth inhibition, A6 was selected for further mechanistic studies.

### 3.2 A6 exerts Significant Anti‐Cancer Activity Against TNBC

**FIGURE 2:**
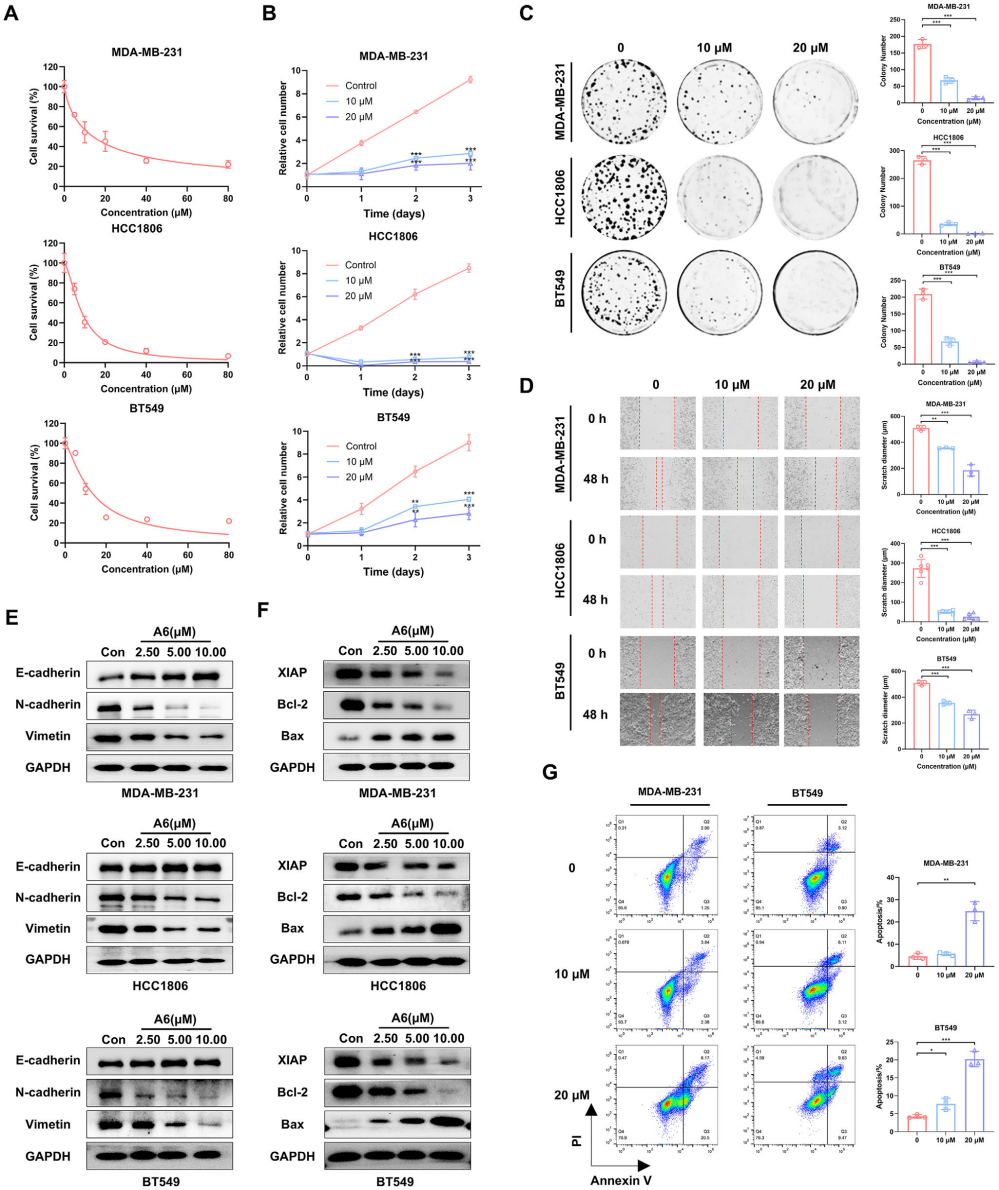
A6 suppresses the malignant phenotypes of TNBC in vitro. (A) MDA‐MB‐231, HCC1806, and BT549 cells were treated with A6 at various concentrations for 72 h. Cell viability was measured using the CCK‐8 assay. Data are presented as mean ± SD (n = 3). (B) MDA‐MB‐231, HCC1806, and BT549 cells were treated with A6 at different time points. The proliferation of cells was determined by cell counting, **p < 0.01, ***p < 0.001. (C) Colony‐formation assay of MDA‐MB‐231, HCC1806, and BT549 cells grown for 12 days in the presence of A6 (10 or 20 µM). Data are presented as mean ± SD (n = 3), ***p < 0.001. (D) Migration of MDA‐MB‐231, HCC1806, and BT549 cells after A6 treatment was measured by wound‐healing assay. Data are presented as mean ± SD (n = 3), **p < 0.01, ***p < 0.001. (E) MDA‐MB‐231, HCC1806, and BT549 cells treated with A6, the levels of E‐cadherin, N‐cadherin, and Vimentin were measured by western blotting. (F) Apoptosis markers such as XIAP, Bcl‐2, and Bax were analyzed by western blotting after A6 treatment. (G) Apoptosis of MDA‐MB‐231 and BT549 cells was determined by flow cytometry after treatment with A6 (10 or 20 µM) for 48 h. Data are presented as mean ± SD (n = 3), *p < 0.05, **p < 0.01, ***p < 0.001.

Further evaluation of A6's anti‐tumor effects revealed that treatment with this compound led to time‐ and dose‐dependent suppression of breast cancer cell proliferation (Figure 2A,B). Consistent with these findings, the colony formation assay demonstrated that A6 significantly reduced both the size and number of colonies compared to the control (Figure 2C). Additionally, the wound‐healing assay revealed that A6 markedly inhibited the migratory capacity of TNBC cells (Figure 2D). Western blotting analysis indicated that A6 suppressed the epithelial‐to‐mesenchymal transition (EMT) pathway (Figure 2E). To investigate the mechanism underlying A6's antiproliferative effects, we examined its ability to induce apoptosis in TNBC cells. As shown in Figure 2F,G, A6 potently triggered TNBC cells apoptosis, as evidenced by increased Bax expression, decreased XIAP and Bcl‐2 levels, and elevated Annexin V staining.

### 3.3 A6 Promotes the Degradation of eEF2K Through the Ubiquitin‐Proteasome Pathway

**FIGURE 3:**
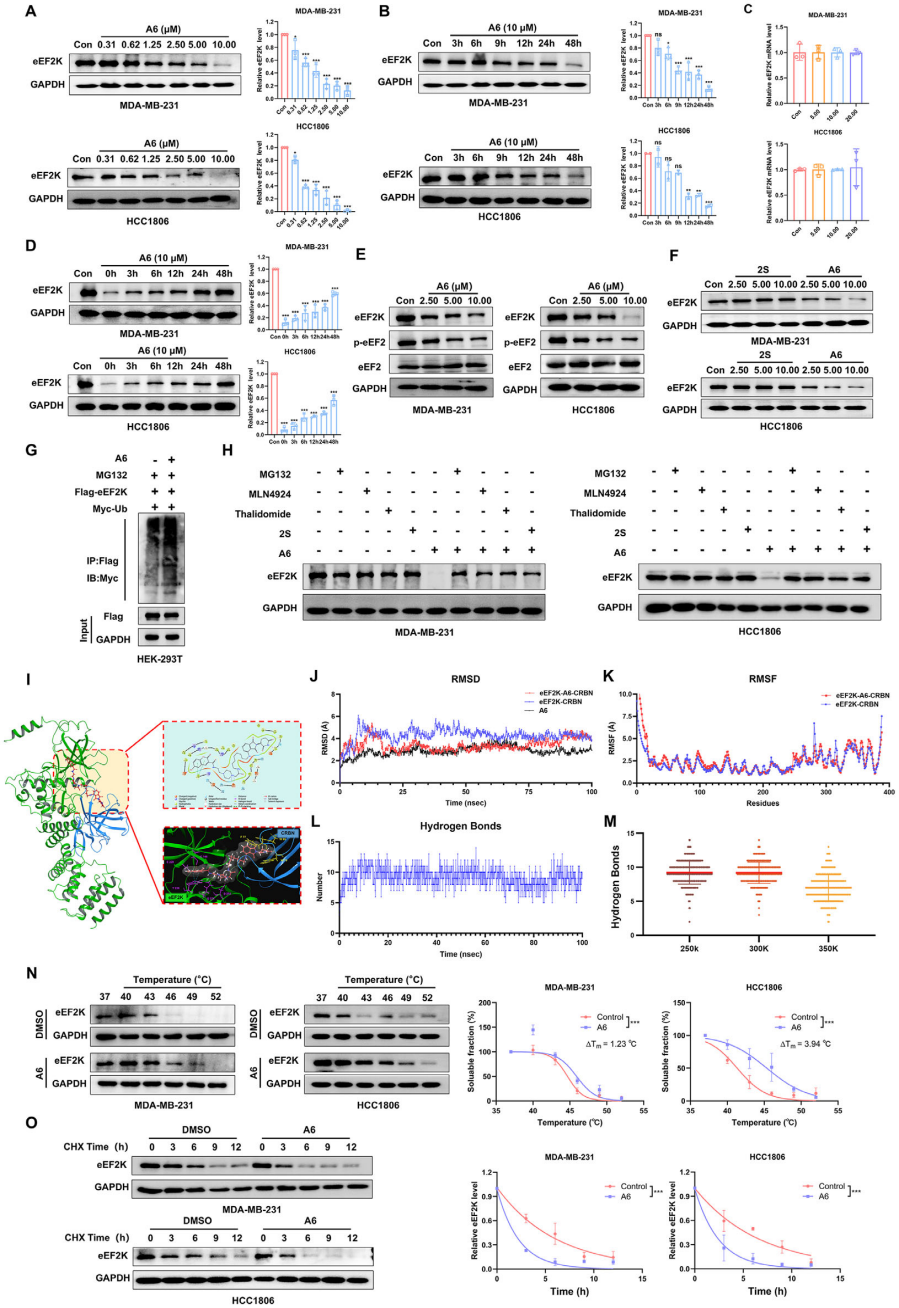
A6 promotes the degradation of eEF2K through the ubiquitin‐proteasome pathway. (A) MDA‐MB‐231 and HCC1806 cells were treated with a series of concentrations of A6 for 48 h, and eEF2K levels were measured by western blotting. Data are presented as mean ± SD; *p < 0.05, ***p < 0.001. (B) MDA‐MB‐231 and HCC1806 cells were treated with A6 for different periods of time, and eEF2K levels were measured by western blotting. Data are presented as mean ± SD; *p < 0.05, **p < 0.01, ***p < 0.001, ns, *p* > 0.05. (C) MDA‐MB‐231 and HCC1806 cells were treated with a series of concentrations of A6, the eEF2K mRNA level was analyzed by real‐time PCR. (D) eEF2K protein recovery after drug washout. (E) MDA‐MB‐231 and HCC1806 cells were treated with a series of concentrations of A6 for 48 h, the levels of eEF2K, phospho‐eEF2(T56) and eEF2 were measured by western blotting. (F) MDA‐MB‐231 and HCC1806 cells were treated with a series of concentrations of A6 or 2S, and eEF2K levels were measured by western blotting. (G) 293T cells were transfected with Flag‐eEF2K and Myc‐Ub plasmid, and then subjected to A6 for 48 h, followed by treatment with MG132 (10 µm) for 4 h before harvest. Then the cell lysates were subjected to immunoprecipitation with anti‐Flag antibodies and blotted with anti‐Myc antibodies. (H) MDA‐MB‐231 and HCC1806 cells were treated with DMSO, thalidomide, 2S, MLN4924 or MG‐132 only; or A6 (10 µM) treated combined with DMSO, thalidomide, 2S, MLN4924 and MG‐132. eEF2K levels were assessed by western blotting. (I) The docking simulation diagram of complex of eEF2K (green, PDB code: 8gm4) with CRBN (blue, PDB code: 4v2z) and A6 (red). (J) The RMSD of A6, eEF2K‐CRBN or eEF2K‐A6‐CRBN. (K) The RMSF of eEF2K‐CRBN or eEF2K‐A6‐CRBN. (L) Hydrogen bonds number during the 100 ns simulation period at the indicated temperature. (M) The number of hydrogen bonds formed in eEF2K‐A6‐CRBN ternary complex in 100 ns at different simulated temperatures. (N) MDA‐MB‐231 cells were treated with or without 10 µM A6 followed by a cellular thermal shift assay (CETSA). Data are presented as mean ± SD, ***p < 0.001. (O) MDA‐MB‐231 and HCC1806 cells were stimulated with 10 µm A6 for 48 h in the presence or absence of 20 µg/mL CHX. eEF2K levels were assessed by western blotting. Data are presented as mean ± SD, ***p < 0.001.

Next, we examined the effect of A6 on the degradation of eEF2K. In TNBC cells, A6 treatment elicited progressive eEF2K depletion, demonstrating clear concentration‐dependent (Figure 3A) and time‐dependent (Figure 3B) degradation kinetics. Quantitative PCR analysis showed no significant changes in eEF2K mRNA levels at the tested concentrations (Figure 3C). Washout experiments demonstrated sustained pharmacodynamic effects, with eEF2K protein levels recovering to <50% of baseline values even 48 h after compound removal (Figure 3D). This prolonged degradation kinetics is characteristic of PROTACs‐mediated catalytic activity, where target elimination persists post‐treatment. Consistent with the inhibition of eEF2K, A6 not only suppressed the phosphorylation of its canonical substrate, eEF2, but also reduced the levels of its recently reported downstream effectors, AURKA and SOX8 [23] (Figure 3E; Figure S1A). These results confirmed the functional impact of eEF2K depletion and demonstrate a broader downstream signaling perturbation. Notably, the parent ligand 2S failed to reduce eEF2K protein levels (Figure 3F), underscoring the fundamental distinction between conventional kinase inhibition and PROTAC‐induced degradation. Mechanistically, A6 significantly enhanced eEF2K polyubiquitination (Figure 3G), leading to its subsequent degradation. This A6‐mediated effect was abrogated by treatment with either the proteasome inhibitor MG132 or E1 inhibitor MLN4924 (Figure 3H). Moreover, pre‐treatment with these inhibitors also blocked the anti‐proliferative function of A6 (Figure S1B,C), indicating that its activity depends on functional proteasome and neddylation pathways.

Computational docking simulations showed that A6 facilitates the formation of a stable ternary complex between eEF2K and CRBN, with key hydrogen bonds reinforcing the eEF2K‐A6‐CRBN interface (Figure 3I). MD simulations further demonstrated that A6 enhances complex stability through persistent interactions with catalytic residues in both eEF2K's allosteric pocket and CRBN's thalidomide‐binding domain (Figure 3J,K). The reference conformation maintained ≥9 hydrogen bonds throughout the 200‐ns trajectory (Figure 3L), with bond counts remaining above seven despite physiological temperature fluctuations (Figure 3M). Cellular thermal shift assays (CETSA) further confirmed direct target engagement, demonstrating that A6 increased eEF2K's thermal stability compared to vehicle controls (Figure 3N). Additionally, cycloheximide chase assays revealed that A6 accelerated eEF2K turnover (Figure 3O). Collectively, these orthogonal approaches establish A6 as a bona fide eEF2K‐targeting PROTAC degrader.

### 3.4 A6 Exhibits Potent Anti‐Tumor Activity Against TNBC In Vivo and in TNBC organoid models

**FIGURE 4:**
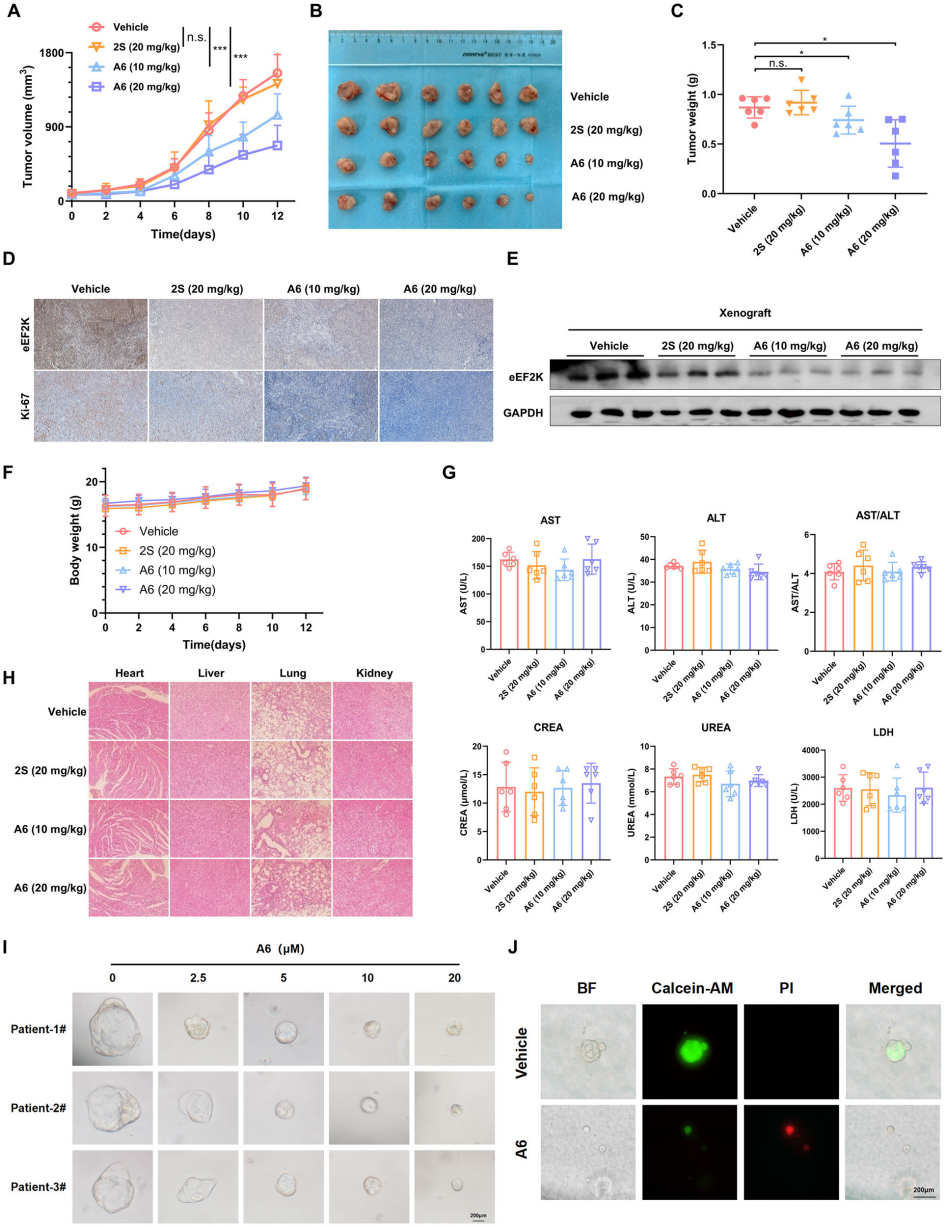
A6 suppresses the malignant phenotypes of TNBC in vivo and in TNBC organoid models. Mice bearing MDA‐MB‐231 xenografts were administered vehicle (DMSO), 2S (20 mg/kg), and A6 (10 or 20 mg/kg), with tumor volumes measured every other day. (A)Tumor growth curve is shown. ***p < 0.001, ns, *p* > 0.05. (B) Image of resected xenografts. (C) Tumor weight from xenografts, *p < 0.05, ns, *p* > 0.05. (D) Representative immunohistochemical (IHC) staining of eEF2K and Ki‐67. (E) eEF2K levels in tumors were measured by western blotting. (F) Body weights of mice. (G) Serum biochemical parameters for liver and kidney were measured for each animal treated with vehicle control, 2S, or A6. (H) Organs were subjected to hematoxylin and eosin (H&E) staining, and representative images are shown. (I) Representative images of TNBC patient‐derived organoids after treatment by A6 (Scale bar 200 µm). (J) The growth inhibition of A6 was demonstrated by Calcein‐AM/PI fluorescence staining of PDO (Scale bar 200 µm).

Building on the strong in vitro antiproliferative and degradation effects of A6, we further evaluated the antitumor activity of the compound in vivo using BALB/c nude mice bearing MDA‐MB‐231 xenograft tumors. Intraperitoneal administration of A6 at 10 mg/kg or 20 mg/kg significantly suppressed tumor growth. In contrast, compound 2S showed only a minimal effect on tumor growth (Figure 4A–C). These finding were further supported by a reduction in Ki‐67 expression (Figure 4D). Additionally, we assessed eEF2K protein levels in xenograft tumor tissues. Immunohistochemical and Western blot analysis revealed a substantial decrease in eEF2K expression in A6‐treated tumors compared to the vehicle group (Figure 4D,E). Notably, A6 was well‐tolerated in mice, with no significant body weight loss observed at either the 10 mg/kg or 20 mg/kg dose (Figure 4F). Serum biochemical analysis showed no notable abnormalities in markers of liver and kidney function, suggesting an absence of hepatorenal toxicity (Figure 4G). Furthermore, histological examination via H&E staining revealed no obvious morphological damage in major organs, including the heart, liver, lungs, and kidneys (Figure 4H).

To further validate the antitumor efficacy of A6, we employed a patient‐derived TNBC organoid (PDO) model. As shown in Figure 4I,J, A6 exerted a pronounced inhibitory effect on TNBC, as evidenced by a reduction in organoid formation and an increase in cell death indicated by live/dead staining. These results collectively demonstrate that A6 exerts potent anti‐cancer efficacy against TNBC both both in vitro and in vivo, accompanied by a favorable safety profile.

### 3.5 The Anti‐Cancer Activity of A6 is Critically Dependent on Both CRBN and eEF2K

**FIGURE 5:**
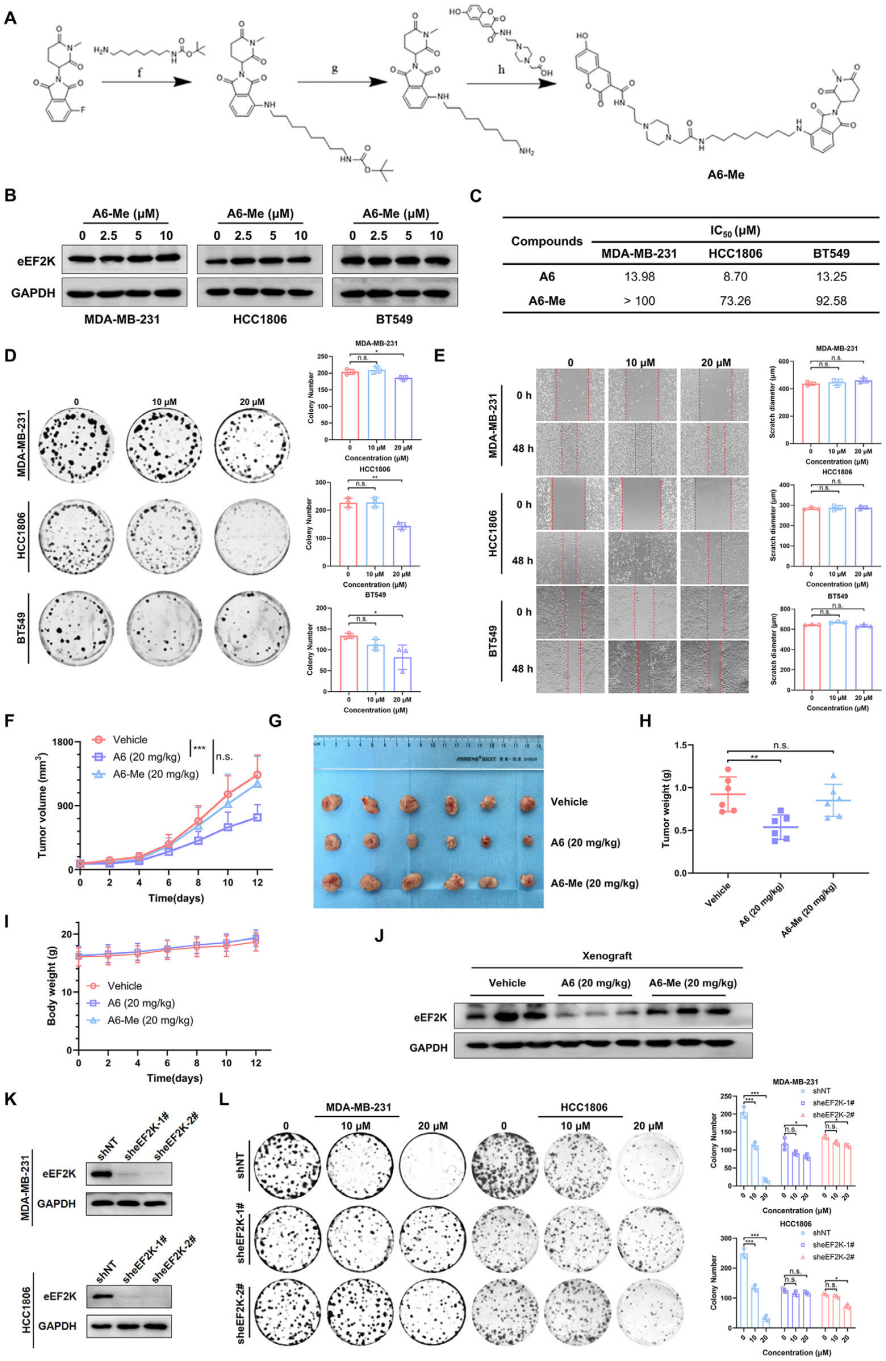
The anti‐cancer activity of A6 is critically dependent on both CRBN and eEF2K. (A) Synthesis of A6‐Me. Reagents and conditions. (B) Western blotting analysis of eEF2K expression in MDA‐MB‐231, HCC1806, and BT549 cells treated with A6‐Me for 48 h. (C) IC_50_ values of A6 and A6‐Me in TNBC cells. (D) Colony‐formation assay of MDA‐MB‐231, HCC1806, and BT549 cells grown for 12 days in the presence of DMSO and A6‐Me (10 or 20 µM). Data are presented as mean ± SD (n = 3). *p < 0.05, **p < 0.01, ns, *p* > 0.05. (E) Migration of MDA‐MB‐231, HCC1806, and BT549 cells after A6‐Me treatment was measured by wound‐healing assay. Data are presented as mean ± SD (n = 3), ns, *p* > 0.05. (F) Mice bearing MDA‐MB‐231 xenografts were administered vehicle (DMSO), A6 (20 mg/kg), and A6‐Me (20 mg/kg), and tumor volumes were measured every other day. Tumor growth curve was shown. ***p < 0.001, ns, *p* > 0.05. (G) Image of resected xenografts. (H) Tumor weight of xenografts, **p < 0.01, ns, *p* > 0.05. (I) Body weight of mice. (J) eEF2K levels in tumors were measured by western blotting. (K) Western blotting was used to examine eEF2K expression in non‐target (shNT) or stable eEF2K knockdown (sheEF2K 1#, 2#) in MDA‐MB‐231 and HCC1806 cells. (L) Colony‐formation assay for proliferation of control and eEF2K‐KD cells treated with A6. Data are presented as mean ± SD (n = 3). *p < 0.05, ***p < 0.001, ns, *p* > 0.05.

To further investigate the functional relevance of CRBN binding and eEF2K degradation in A6's anti‐cancer activity, we modified the glutarimide moiety‐a key structural element known to mediate interaction with the CRBN E3 ligase. Previous studies have shown that methylation of this disrupts CRBN binding and impairs degradation efficiency [38]. Based on this, we synthesized a methylated derivative of A6, designated A6‐Me (Figure 5A). As expected, A6‐Me exhibited significantly reduced efficacy in degrading eEF2K compared to A6 (Figure 5B). It also showed 8‐ to 10‐fold lower potency in inhibiting the viability of MDA‐MB‐231 and HCC1806 breast cancer cells (Figure 5C). Colony formation assays further demonstrated that A6‐Me had only minimal effects on cell proliferation (Figure 5D), and it was significantly less effective than A6 in inhibiting cell migration (Figure 5E). Consistent with the in vitro results, A6‐Me showed markedly weaker antitumor effects in vivo compared to A6 (Figure 5F–I), with no detectable reduction in eEF2K levels in tumor tissues (Figure 5J). These results underscore the necessity of efficient CRBN engagement for eEF2K degradation, and antitumor efficacy.

To further validate that the antitumor effects of A6 tumor inhibitory are mediated specifically through eEF2K targeting, we generated stable eEF2K‐knockdown (KD) MDA‐MB‐231 and HCC1806 cells using eEF2K shRNA (Figure 5K). The eEF2K‐KD cells exhibited significantly reduced sensitivity to A6 treatment compared control cells (Figure 5L), indicating that A6's antitumor activity is highly dependent on eEF2K expression. In conclusion, these findings demonstrate that the antitumor efficacy of A6 relies on the specific downregulation of eEF2K protein levels, highlighting its potential as a targeted therapeutic strategy in breast cancer.

### 3.6 Characterization of the A6‐Loaded Nanoparticles

**FIGURE 6:**
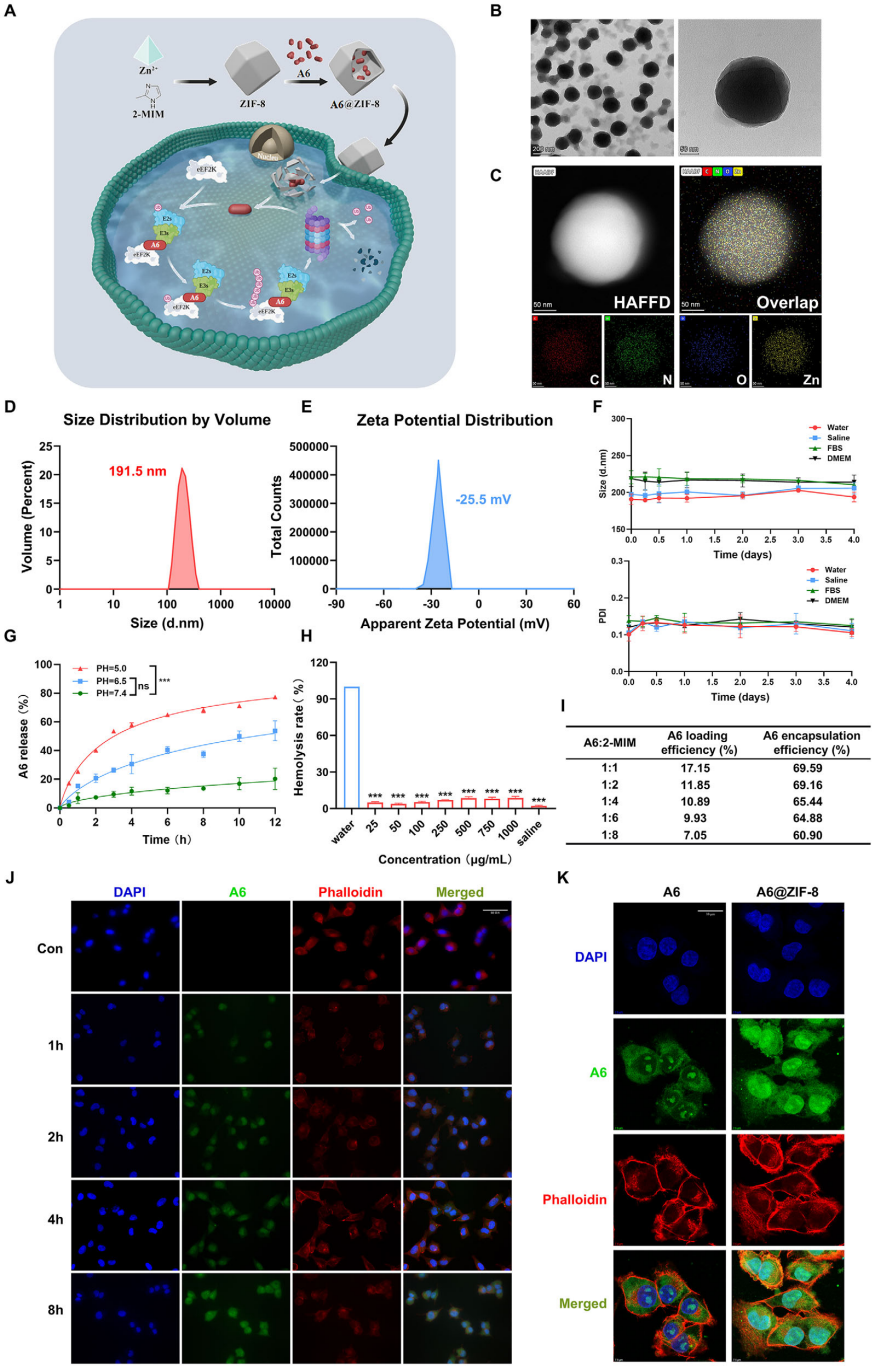
Characterization of A6 nanoparticles. (A) The preparation process of A6@ZIF‐8 and its detailed mechanism for enhanced anticancer therapy. (B) TEM images of A6@ZIF‐8 nanoparticles. (C) Energy‐dispersive X‐ray spectroscopy (EDS) images of A6@ZIF‐8 nanoparticles. (D) Particle size of A6@ZIF‐8. (E) Zeta potential of A6@ZIF‐8. (F) Storage stability investigation of A6@ZIF‐8 (n = 3). (G) Release of A6 from A6@ZIF‐8 nanoparticles in PBS solutions (pH = 5.0, pH = 6.5, or pH = 7.4). (H) Hemolysis test of A6@ZIF‐8 nanoparticles at different concentrations, ***p < 0.001. (I) Effects of A6 concentration on particle size, distribution coefficient, encapsulation rate, and drug loading of A6@ZIF‐8. (J) Fluorescent images of MDA‐MB‐231 cells after incubation with A6@ZIF‐8 NPs for different time points (Scale bar 50 µm). (K) Confocal laser scanning microscope (CLSM) images of MDA‐MB‐231 cells incubated with A6 or A6@ZIF‐8 NPs for 2 h (Scale bar 10 µm).

Previous studies have successfully developed active‐targeted nanomedicines based on MOFs for the in vivo targeted delivery of PROTACs, offering a promising strategy to enhance tumor therapy [39, 40]. Building on the antitumor efficacy of A6, we developed a nanocarrier system for its targeted delivery to tumor sites (Figure 6A). Transmission Electron Microscope (TEM) images revealed that the NPs maintained a well‐defined dodecahedral morphology, confirming their core‐shell structure (Figure 6B). Energy Dispersive Spectrometer (EDS) detected the presence of C, O, N, and Zn elements, verifying the composition of the NPs (Figure 6C). The resulting A6@ZIF‐8 NPs exhibited an average particle size of approximately 190 ± 10 nm and a zeta potential of ‐25.5 mV (Figure 6D,E). The colloidal stability of A6@ZIF‐8 NPs was further assessed in various media, including pure water, saline, fetal bovine serum, and DMEM with 10% fetal bovine serum. The NPs remained stable with no significant changes in size over four days, indicating excellent stability (Figure 6F). The drug release profile of A6@ZIF‐8 NPs was examined under different pH conditions. At neutral (pH 7.4), drug release was slow; however, under acidic conditions (pH 5.0), which simulate the Tumor Mircroenvironment (TME), 77% of A6 was released, demonstrating strong pH‐responsive properties of the system (Figure 6G). In vitro hemolysis assays revealed that A6@ZIF‐8 NPs exhibited a hemolysis rate of 10%, with no significant hemolytic phenomenon observed, thus verifying their satisfactory in vitro blood biocompatibility (Figure 6H). The encapsulation efficiency (EE) and drug loading content (DL) of A6 in ZIF‐8 were optimized by varying the A6:2‐methylimidazole (2‐MIM) molar ratios (1:1, 1:2, 1:4, 1:6, and 1:8) (Figure 6I). Quantified by HPLC, A6@ZIF‐8 prepared at an A6:2‐MIM ratio of 1:1 achieved the highest EE (69.6%), corresponding to a DL of 17.2% (w/w). Notably, A6 encapsulation was relatively insensitive to the 2‐MIM molar ratio, suggesting that A6 incorporation occurred mainly during the early nucleation/co‐precipitation stage of ZIF‐8 formation. Therefore, the 1:1 formulation was selected for subsequent studies. Fluorescence microscopy revealed that while free A6 had limited ability to cross the cell membrane, A6 encapsulated within A6@ZIF‐8 NPs was efficiently internalized within 2 h, as evidenced by strong green fluorescence signals (Figure 6J,K). These results confirm the superior cellular uptake and effective intracellular delivery of A6 mediated by the ZIF‐8 NP system.

### 3.7 Nanoparticle‐Mediated Delivery of A6 Exerts Strong Anti‐Tumor Effects on TNBC Cells and Xenograft Models

**FIGURE 7:**
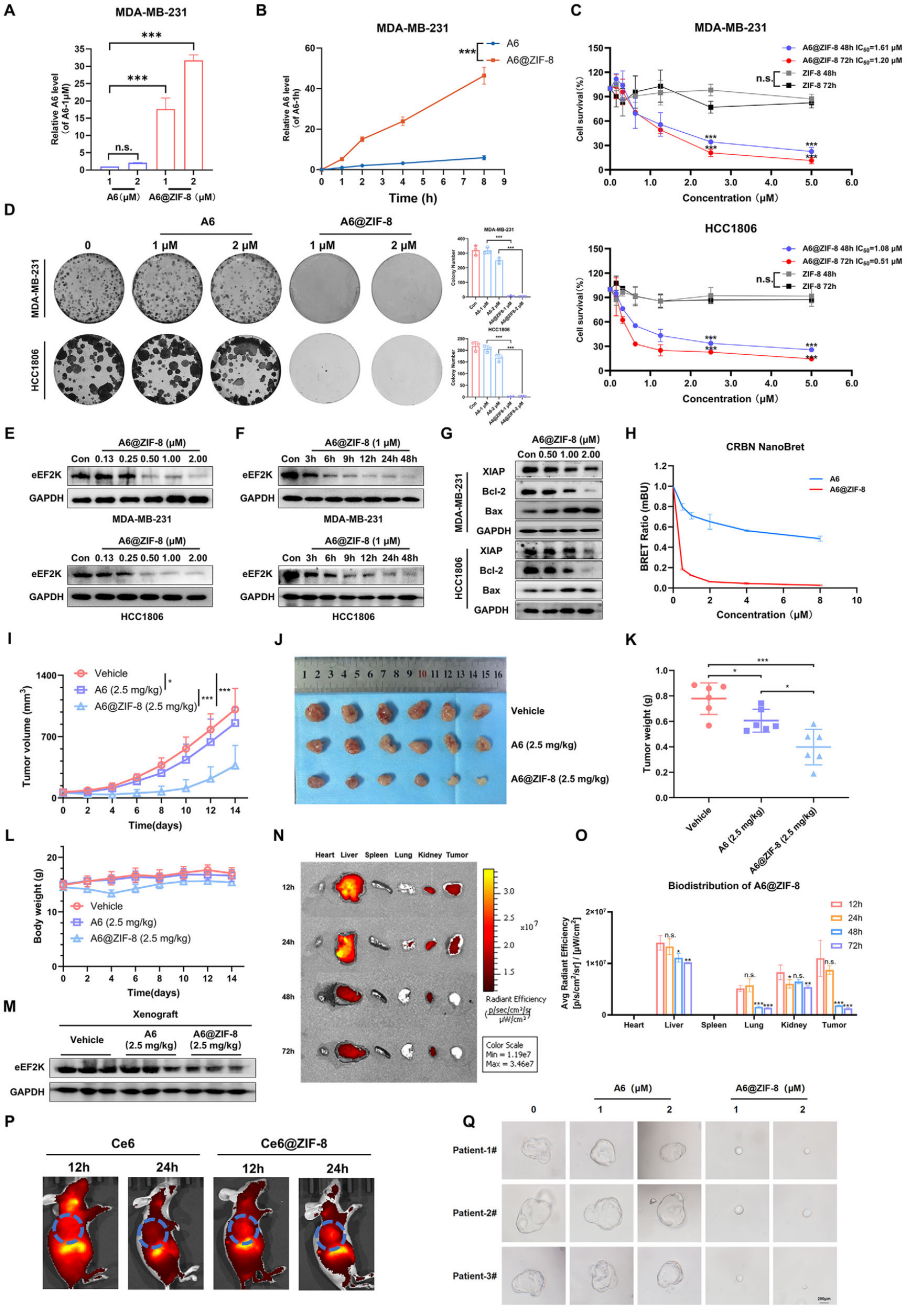
Nanoparticle‐mediated delivery of A6 exerts strong anti‐tumor effects on TNBC cells and xenograft models. (A) Intracellular accumulation of A6 and A6@ZIF‐8 in MDA‐MB‐231 cells (n = 3), with A6 at 1 µM used as a loading control, ***p < 0.001, ns, *p* > 0.05. (B) Time‐dependent intracellular accumulation of A6 and A6@ZIF‐8 (1 µM) in MDA‐MB‐231 cells (n = 3). The sample from cells treated with A6 for 1 h was used as a loading control, ***p < 0.001. (C) MDA‐MB‐231 and HCC1806 cells were treated with A6, A6@ZIF‐8, and ZIF‐8 at various concentrations for 48 or 72 h, followed by CCK‐8 assay, ***p < 0.001, ns, *p* > 0.05. (D) Colony‐formation assay of MDA‐MB‐231 and HCC1806 cells grown for 12 days in the presence of A6 and A6@ZIF‐8. Data are presented as mean ± SD (n = 3), ***p < 0.001. (E) Western blotting analysis of eEF2K expression in MDA‐MB‐231 and HCC1806 cells treated with A6@ZIF‐8 at different concentrations for 48 h. (F) Western blotting analysis of eEF2K in MDA‐MB‐231 and HCC1806 cells treated with 1 µM of A6@ZIF‐8 for the indicated time points. (G) Apoptosis markers such as XIAP, Bcl‐2, and Bax were analyzed by Western blotting after treatment by A6@ZIF‐8. (H) NanoBRET kinetic degradation assay: BromoTag–HiBiT‐CRBN knock‐in HEK293T cells were treated with A6 or A6@ZIF‐8 at different concentrations for 3 h (n = 3). (I) Mice bearing MDA‐MB‐231 xenografts were administered vehicle, A6 (2.5 mg/kg), or A6@ZIF‐8 (2.5 mg/kg), and tumor volumes were measured every other day. Tumor growth curve is shown, *p < 0.05, ***p < 0.001. (J) Image of resected xenografts. (K) Tumor weight from xenografts, *p < 0.05, ***p < 0.001. (L) Body weights of mice shown. (M) eEF2K levels in tumors were measured by western blotting. (N‐O) Representative ex‐vivo fluorescence images and quantitative fluorescence intensity of tumors and organs collected from mice at 12, 24, 48, and 72 h post‐injection (n = 3 mice per group, *p < 0.05, **p < 0.01, ***p < 0.001, ns, *p* > 0.05). (P) In Vivo fluorescence images of mice were taken at indicated time points postinjection. (Q) Representative images of TNBC patient‐derived organoids after treatment by A6 or A6@ZIF‐8 (Scale bar 200 µm).

Next, we evaluated the inhibitory effects of A6@ZIF‐8 nanoparticles on TNBC. The intracellular accumulation of A6 delivered via NPs was first assessed. A6@ZIF‐8 treatment led to a rapid and marked enhancement of intracellular A6 accumulation relative to free A6 (Figure 7A,B), demonstrating the superior cellular delivery efficiency of the nano‐formulation. Consistent with this result, A6@ZIF‐8 showed stronger cytotoxicity against cancer cells than free A6 (Figure 7C,D). Furthermore, the DC_50_ of A6@ZIF‐8 for eEF2K in HCC1806 cells was approximately 68 nM, with nearly complete degradation of eEF2K observed at 1 µM, indicating that nano‐encapsulation markedly improves the degradation efficiency of A6 (Figure 7E,F). Apoptosis‐related markers, including Bcl‐2, Bax, and XIAP, were also modulated following A6@ZIF‐8 treatment (Figure 7G), further supporting the enhanced antitumor effect of mediated by the nanoparticle delivery system. Using Nano‐BRET assays, we observed that the nanoformulation significantly enhances the rate of A6 ternary complex formation (Figure 7H). We further investigated the therapeutic potential of A6@ZIF‐8 in vivo. As shown in Figure 7I–K, A6@ZIF‐8 exhibited significantly stronger efficacy in MDA‐MB‐231 xenograft models. Notably, no significant difference in body weight were observed across the treatment groups (Figure 7L), suggesting good tolerability at the administered dose. Moreover, A6@ZIF‐8 treatment induced substantial degradation of eEF2K in tumor tissues (Figure 7M). In biodistribution studies, we administered ZIF‐8 nanoparticles loaded with the near‐infrared dye Ce6 via intravenous (i.v.) injection. Consistent with our design, significant tumor‐specific accumulation of A6@ZIF‐8 was observed, followed by systemic clearance within 48 h post‐injection. (Figure 7N‐P). In addition, patient‐derived TNBC organoids further confirmed that A6@ZIF‐8 significantly suppressed TNBC growth, with a stronger inhibitory effect than A6 (Figure 7Q). In conclusion, these results demonstrate that the ZIF‐8‐based nanodelivery systems significantly enhances the practical efficacy of PROTAC compounds for cancer therapy.

## 4 Discussion

Mounting clinical and preclinical evidence has established eEF2K as a critical therapeutic target in breast cancer subtypes and other malignancies, with its overexpression strongly correlated to disease progression and therapy resistance [6]. However, pharmacologically targeting eEF2K remains challenging: ATP‐competitive inhibitors often lack sufficient selectivity, while allosteric binders targeting the non‐catalytic pocket, though more specific, exhibit suboptimal therapeutic efficacy [24]. The emergence of TPD technologies, particularly PROTACs and molecular glues, has revolutionized therapeutic development by enabling precise elimination of pathologically hyperactivated proteins, including those traditionally deemed “undruggable” [41, 42]. Our team previously developed eEF2K degraders to inhibit malignant phenotype in TNBC [20, 22, 23]. Given the superior druggability of PROTACs, we employed this technology to overcome the challenges associated with eEF2K inhibition. In this study, a series of compounds were designed and synthesized as potent eEF2K degraders, identifying A6 induces eEF2K degradation via the ubiquitin‐proteasome pathway. The antiproliferative effect of A6 on cancer cells is eEF2K‐dependent; loss of degradation capacity markedly attenuated its antitumor efficacy. Compared to previously reported eEF2K‐targeting PROTACs [32], A6 offers notable advantages. Earlier compounds lacked comprehensive biological and pharmacological profiling, as well as in vivo anticancer validation. In contrast, A6 demonstrates superior pharmacological activity, achieving nanomolar‐range DC_50_ values and over 99% degradation maximum (DCmax) in TNBC cells.

PROTAC modalities encompass diverse architectural designs, including peptide‐based constructs, small‐molecule degraders, and E3 ligase‐recruiting systems utilizing MDM2, VHL, or CRBN [43]. Among these, CRBN‐based PROTACs have shown particular promise in targeting drug‐resistant cancers [44]. A6 incorporates a CRBN‐binding motif, and our findings highlight the critical role of this interaction in mediating efficient eEF2K degradation and enhancing antitumor activity. These results suggest that A6 could play a pivotal role in overcoming drug resistance in cancer treatment.

The therapeutic potential of PROTACs is often limited by insufficient tumor specificity, suboptimal pharmacokinetics [45], and low aqueous solubility‐which adversely affects oral bioavailability and membrane permeability [43]. To address these issues, prior studies have explored zeolitic imidazolate framework‐8 (ZIF‐8) as a carrier, combining nanotherapy with chemotherapeutic agents for precise immune targeting and tumor eradication [46]. In this study, we developed a region‐confined PROTAC nanoplatform based on a nanocarrier delivery system (A6@ZIF‐8) for effective degradation of eEF2K. The ZIF‐8 carrier features pH‐responsive degradation, enabling selective release of A6 in the tumor microenvironment. Its high porosity and surface area facilitate efficient loading of hydrophobic PROTAC molecules such as A6. Moreover, the synthesis of A6@ZIF‐8 is mild and rapid, which helps preserve the biological activity of the loaded A6. Following nano‐delivery of A6, its efficacy in degrading eEF2K was significantly enhanced, as evidenced by an approximately 10‐fold decrease in the DC_50_ to 68 nM in HCC1806 cells and significantly enhanced its anticancer potency. This improvement is likely attributable to the nearly 15‐fold increase in intracellular accumulation of A6 mediated by the nano‐delivery system. This work represents a promising strategy for integrating PROTACs with nanotechnology, offering valuable insights for drug development and potential improvements in cancer treatment outcomes.

Despite these encouraging results, several limitations remain. First, enhancing the intracellular stability of PROTACs remains a challenge for clinical translation. Furthermore, although we demonstrated that A6 mediates eEF2K degradation through ternary complex formation, the crystal structure of this complex has not been resolved. Future studies should pursue structural elucidation of CRBN, eEF2K, and their complexes with A6. Additionally structural optimization of A6 is also warranted to improved degradation efficacy and pharmacological properties.

In conclusion, our study presents a TPD‐based strategy targeting eEF2K using A6, a selective degrader demonstrating potent anticancer activity both in vitro and in vivo. Moreover, nanoparticle‐mediated delivery significantly enhanced the degradation efficiency of A6. These findings not only nominate A6 as a potential therapeutic candidate but also provide critical insights for the clinical treatment of breast cancer.

## Author Contributions

S.C., C.Z. and S.J. are co‐first authors and contributed equally to this work. Y.C. and X.Y. designed the study and revised the manuscript. S.C., C.Z. and S.J. performed the experiments, analyzed the experimental data, and drafted the manuscript. Y.X. and M.X. handled the synthesis of NPs. Y.L., T.J. and X.W. analyzed the experimental data. Z.C. provided technical support in the experiments.

## Conflicts of Interest

The authors declare no conflict of interest.

## Supporting information




**Supporting File**: advs73914‐sup‐0001‐SuppMat.pdf.

## Data Availability

The data that support the findings of this study are available in the supplementary material of this article.
