# Supplementary material for: Targeted Degradation of eEF2K by a Structure‐Guided PROTAC Strategy for the Treatment of Triple‐Negative Breast Cancer
Source: Adv Sci (Weinh). 2026 Jan 27;13(18):e20863. doi: 10.1002/advs.202520863 (PMC13042790; doi:10.1002/advs.202520863)
Supplement: Supplementary file 1 — Supporting File: advs73914‐sup‐0001‐SuppMat.pdf. [file ADVS-13-e20863-s001.pdf]

Supporting Information

Targeted Degradation of eEF2K by a Structure-Guided PROTAC Strategy for the Treatment of Triple-Negative Breast Cancer

Shijun Cao*, Changxin Zhong*, Shilong Jiang*, Yungui Li, Yang Xi, Mingxuan Xiao, Ting Jiang, Xiaoya Wan, Zonglin Chen, Xiaohui Yu‡, Yan Cheng‡

File list

Supplementary Figure:

Figure S1 A6 promotes the degradation of eEF2K through the ubiquitin-proteasome pathway.

Supplementary Tables:

Table S1 The IC_{50} values of representative eEF2K-PROTACs in different tumor cell lines

Table S2 The IC_{50} values of A6 in non-TNBC cell lines

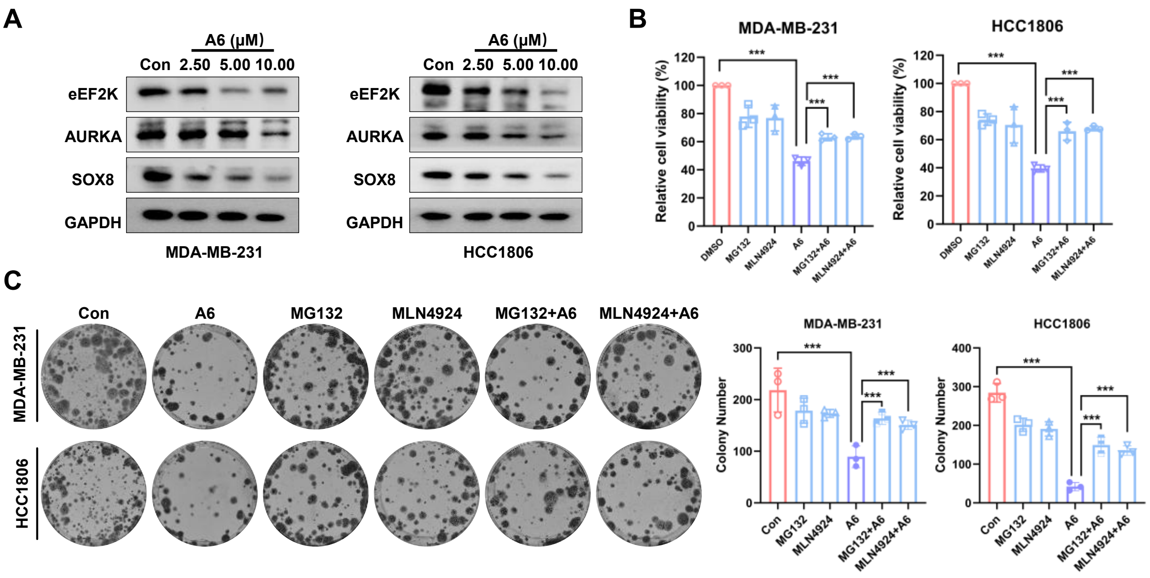

Figure S1 A6 promotes the degradation of eEF2K through the ubiquitin-proteasome pathway. (A) MDA-MB-231 and HCC1806 cells were treated with a series of concentrations of A6 for 48 h, the levels of eEF2K, AURKA and SOX8 were measured by western blotting. (B) MG132 or MLN4924 pre-treatment attenuates the anti-proliferative effects of A6 in MDA-MB-231 and HCC1806 cells, as determined by CCK-8 assay. Data are presented as mean ± SD, $^{***}p < 0.001$. (C) Colony formation ability of MDA-MB-231 and HCC1806 cells following pre-treatment with MG132 or MLN4924 and subsequent exposure to A6. Data are presented as mean ± SD, $^{***}p < 0.001$.

Table S1 The IC_{50} values of representative eEF2K-PROTACs in different tumor cell lines

Compounds	IC_{50} (μM)	
	MDA-MB-231	HCC1806
A5	32.95	12.25
A6	13.98	8.70
A7	30.27	14.74
B5	82.35	> 100
B6	> 100	> 100
B7	40.08	33.04
2S (ligand)	> 100	> 100

Table S2 The IC_{50} values of A6 in non-TNBC cell lines

Compounds	IC_{50} (μM)			
	MCF-7	T47D	SK-BR-3	MCF-10A
A6	26.28	29.7	30.01	> 100
